# An Artificial PVA-BC Composite That Mimics the Biomechanical Properties and Structure of a Natural Intervertebral Disc

**DOI:** 10.3390/ma15041481

**Published:** 2022-02-16

**Authors:** Mengying Yang, Dingding Xiang, Yuru Chen, Yangyang Cui, Song Wang, Weiqiang Liu

**Affiliations:** 1 Department of Mechanical Engineering, Tsinghua University, Beijing 100084, China; ymy18@mails.tsinghua.edu.cn (M.Y.); chenyr19@mails.tsinghua.edu.cn (Y.C.); cuiyy20@mails.tsinghua.edu.cn (Y.C.); 2 State Key Laboratory of Tribology, Tsinghua University, Beijing 100084, China; 3 Tsinghua Shenzhen International Graduate School, Tsinghua University, Shenzhen 518055, China; 4 Biomechanics and Biotechnology Lab, Research Institute of Tsinghua University in Shenzhen, Shenzhen 518057, China; 5 School of Mechanical Engineering and Automation, Northeastern University, Shenyang 110819, China

**Keywords:** PVA-BC, intervertebral disc, biomechanics, creep, unconfined compression

## Abstract

Disc herniation is one of the most ubiquitous healthcare problems in modern cities—severe patients eventually require surgical intervention. However, the existing operations—spinal fusion and artificial disc replacement—alter the biomechanics of the spine, leaving much room for improvement. The appropriateness of polyvinyl alcohol (PVA) for biomedical applications has been recognised due to its high water content, excellent biocompatibility, and versatile mechanical properties. In this study, a newly-designed PVA–bacterial cellulose (PVA-BC) composite was assembled to mimic both the biomechanics and annular structure of natural intervertebral discs (IVDs). PVA-BC composites of various concentrations were fabricated and tested under unconfined compression and compressive creep in order to acquire the values of the normalised compressive stiffness and whole normalised deformation. The normalised compressive stiffness increased considerably with an increasing PVA concentration, spanning from 1.82 (±0.18) to 3.50 (±0.14) MPa, and the whole normalised deformation decreased from 0.25 to 0.13. Formulations of 40% PVA provided the most accurate mimicry of natural human IVDs in normalised whole deformation, and demonstrated higher dimensional stability. The biocompatible results further confirmed that the materials had excellent biocompatibility. The novel bionic structure and formulations of the PVA-BC materials mimicked the biomechanics and structure of natural IVDs, and ensured dimensional stability under prolonged compression, reducing the risk of impingement on the surrounding tissue. The PVA-BC composite is a promising material for third-generation artificial IVDs with integrated construction.

## 1. Introduction

Disc herniation is one of the most common health problems troubling millions of people in modern cities. It is principally caused by degenerative disease or the unexpected injury of intervertebral discs (IVDs). For severe patients, eventual surgical interventions are required, including spinal fusion and total disc replacement (TDR) [1,2]. Fusion, the gold standard for treating degenerative disc disease (DDD), aims to eradicate pain by eliminating the motion of the treated segment while restoring the height of the normal disc with implanted instrumentation. However, complications of adjacent segment diseases and loss of motion have urged an alternative treatment of TDR that preserves the motion of the treated segment and decompresses using a dynamic device [3,4,5]. Popular devices such as CHARITÉ (DePuy Spine, Inc., Raynham, MA, USA), Prodisc (Synthes, Inc., West Chester, PA, USA), and Activ (Aesculap Implant Systems, LLC, Center Valley, PA, USA) are artificial disc prostheses that replace natural IVDs with the structure of an articulating ball-and-socket joint. Unfortunately, they introduce the inevitable problems of friction and wear [6,7]. Moreover, in most clinical studies, the outcomes of TDR were not superior to fusion [8,9,10,11,12].

Several second-generation artificial discs have been proposed with more biomimetic designs [13], including the Freedom disc (Axiomed, Cleveland, OH, USA), the Bryan disc (Medtronic, Minneapolis, MN, USA), and the M6 artificial disc (Spinal Kinetics, Sunnyvale, CA, USA) [14,15]. However, these devices failed to abandon the metal endplates. They may limit the range of motion (ROM) and hamper follow-up imaging due to metal artefacts, particularly in complicated cervical segments [16,17].

These unsatisfactory clinical results from surgical interventions are due primarily to the unsuitable selections of structures and biomaterials of existing prostheses, altering the biomechanics of spinal segments. With fusion and TDR, the load and motion are transmitted by the rigid connection and the articulating contact, whereas natural IVDs are deformable soft tissue in which the load and motion are provided by viscoelasticity [18,19,20]. From the perspective of bionic optimisation, these problems may be addressed by providing a viscoelastic “cushion” that includes axial compliance instead of rigid connection, preventing the prosthesis from sinking in medium- and long-term clinical practice. The appropriateness of PVA for biomedical applications has been recognised in terms of its high water content, excellent biocompatibility, and versatile mechanical properties in the areas of artificial cartilage [21,22,23,24] and knee meniscus [25,26,27,28,29]; it has demonstrated a potential use in nucleus pulposus (NP) replacements [30,31,32] and TDR [33,34].

By mimicking the annular structure of natural IVDs, natural equivalent properties are expected to be duplicated in the novel PVA-BC composite, thus help in reconstructing functions of total discs. The natural IVD is formed by a gel-like and deformable NP surrounded by multiple layers of concentric fibres. The NP principally consists of water (70–90% by weight) [35,36,37], proteoglycan (35–65% of the dry weight of the NP) [38,39,40], and fine collagen type-II fibrils (5–20% of the dry weight of the NP) [41,42]. The fine collagen fibrils provide a three-dimensional network in which proteoglycan is trapped, resulting in an osmotic pressure that resists compression. The annulus fibrosus (AF) consists of 15–25 concentric layers which are 0.05–0.5 mm thick, and contains water (65–70% by weight), proteoglycan (20% of the dry weight of the AF), and coarse collagen type I fibre bundles (50–70% of the dry weight of the AF) [43,44,45,46]. Mechanically, type I collagen provides strength in tension, holding the NP together and bonding to the adjacent vertebral bodies (VBs).

PVA hydrogel, as the primary matrix of the composite, may provide elastic restoring force and viscoelastic energy dissipation [47,48,49], which are major functions of natural NP, and can be infiltrated into the outer BC layers to form a double network. BC fibres as the outer layers may mimic the function of natural AF, increasing the tensile strength by sharing the load in the composite framework [50,51]. Furthermore, an additional immersion in 2-acrylamido-2-methylpropane sulfonic acid (AMPS) solution may help to provide negative charges from the sulfate groups on the AMPS molecules, endowing the inner core an osmotic pressure that swells to bear compressive loads.

This study introduces a novel PVA-BC composite for third-generation artificial IVDs, mimicking the structures and biomechanics of AF and NP, anatomically and functionally. The novel design comprises a PVA-based gelatinous core that carries negative charges to preserve swelling after being treated with AMPS and BC multilayers outside that hold the nucleus together and provide tensile strength. The goals of the study were (1) to evaluate the mechanical outcomes for the optimisation of the formulations of the PVA-BC composite, and (2) to assess the cytotoxicity of the novel material for safety assurance for further implanted use.

## 2. Materials and Methods

### 2.1. Fabrication Process

**Figure 1:**
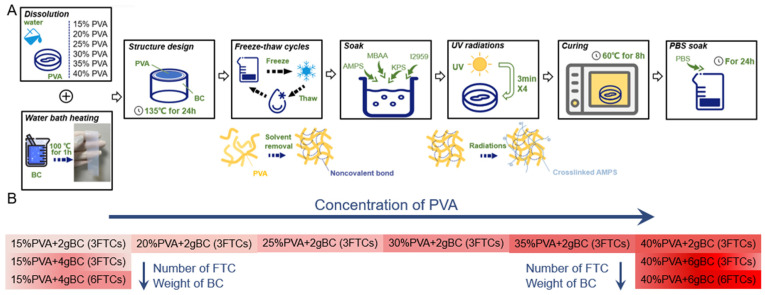
(**A**) Illustration of the PVA-BC composite fabrication process. (**B**) Formulations for the PVA-BC composite.

The PVA-BC composite was fabricated following the path depicted in Figure 1A. First, aqueous solutions of 15, 20, 25, 30, 35, and 40 wt.% PVA (PVA 124, Macklin Biochemical Co., Ltd., Shanghai, China) were prepared. Next, the membrane of bacterial nanocellulose (Bacterial nanocellulose membrane 32 * 26 * 0.3 cm, Qi Hong Technology, Guilin, Guangxi Province, China) was cut into strips, then softened by boiling in 100 °C water for 1 h. The BC multilayers were placed on the inner wall of the hydrothermal reactor, and the PVA aqueous solution was poured into the middle to mimic the natural structure of IVDs. The melt and mutual infiltration were conducted at 135 °C for 24 h, after which the gel was frozen at −80 °C for 1 h and thawed at 4 °C for 2 h, which was one freeze–thaw cycle (FTC), in order to physically crosslink the PVA.

The PVA-BC composite was then soaked in a solution of 60 × 10^−3^ M methylene diacrylamide (MBAA), 0.5 mg mL^−1^ potassium persulfate (KPS), 50 × 10^−3^ M I2959, and 30 wt.% AMPS for 24 h, followed by radiation under UV lights for 12 min. Finally, curing at 60 °C for 8 h ensured the even mechanical properties of the composite. Moreover, the PVA-BC composite required an extra bath in 0.15 M phosphate-buffered saline (PBS) for 24 h before testing. The PVA-BC composite was finally formed with a diameter of 30 mm and a height of 20 mm, and the average mass was 15.91 (± 0.74) g. The formulations are presented in Figure 1B.

### 2.2. Material Property Characterisations

**Figure 2:**
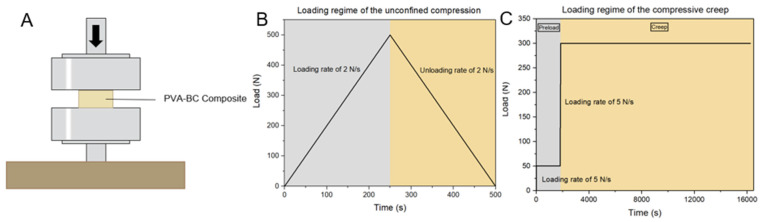
(**A**) Diagram of the mechanical test. (**B**) Loading regime of the unconfined compression. (**C**) Loading regime of the compressive creep.

Each formulation of the PVA-BC composite underwent unconfined compression and compressive creep tests to determine the sensitivity of the mechanical properties to those parameters (the PVA concentration, number of FTCs, and weight of BC). The mechanical tests were conducted at room temperature using the MTS-CMT 6104 (MTS System [China] Co., Ltd.). During the tests, the specimen was placed between platens (Figure 2A), and the results were recorded for comparison with natural IVD values in the published literature.

The unconfined compression was performed in the load-controlled mode at a loading rate of 2 N/s, up to the final force of 500 N (Figure 2B), which is equal to 0.71 MPa. The specimen was then unloaded at an unloading rate of −2 N/s.

The compressive creep provides the basic mechanical properties of the PVA-BC composite because it simulates the most common loading condition of the spine: prolonged sitting or standing [52,53]. The specimen was first preloaded with 50 N for 30 min, then increased to 300 N at 5 N/s for 4 h (Figure 2C), which is equal to 0.425 MPa and corresponds to a human’s bodyweight at 750 N divided by the 1765 mm^2^ disc area [54]. The displacement and load were recorded at 1 Hz, and the step displacement, creep displacement, and compressive stiffness were calculated and compared with natural IVDs.

### 2.3. Data Analysis

Data from the end of the preload to the point reaching 300 N in the compressive creep tests were analysed in order to obtain the compressive stiffness. The tangent values of the load-displacement curves from 50 to 300 N represented the compressive stiffness, and the step displacement was defined as the change in displacement during this period. The creep displacement was the change in the displacement at a constant load of 300 N for 4 h.

The area and height of the PVA-BC composite were used to calculate the normalised values and eliminate the effects of geometric parameters on the above mechanical param-eters. The normalised compressive stiffness was calculated with Equation (1), where *H* is the height of specimens and *A* is the area of the cross-section of specimens. The normalised creep and step displacements were calculated with Equations (2) and (3).

(1) $$Normalized\;compressive\;stiffness=Compressive\;stiffness\times \frac{H}{A}$$

(2) $$Normalized\;creep\;displacement=\frac{Creep\;displacement}{H}$$

(3) $$Normalized\;step\;displacement=\frac{Step\;displacement}{H}$$

### 2.4. Assessment of Cytotoxicity

For the evaluation of the cytotoxicity of the PVA-BC composite, 0.6 g material was immersed in 3 mL solution of minimum essential medium (MEM), 10% fetal bovine serum (FBS), and 1% antibiotics (penicillin and streptomycin) for 24 h in a 37 ℃ oscillator to prepare the leach liquor. Three groups were established: the blank MEM control group, the positive dimethyl sulfoxide (DMSO) group, and the trial group containing the leach liquor and MEM. Next, the L-929 mouse fibroblasts were diluted to a concentration of 1.5 × 10^4^ cell/well in a 96-well cell culture plate, and were then cultured with the three groups of solutions for one day. On day 2, the absorbance values of the three groups, containing six wells each, were assessed and recorded using an enzyme-labelled instrument (iMark, Bio-Rad Laboratories Inc., Hercules, CA, USA) with a 450-nm detection filter.

### 2.5. Statistical Analysis

A statistical analysis was performed on the normalised compressive stiffness and the whole normalised deformation using single-factor analysis of variance (ANOVA), where the factor was the formulations. All of the statistical analyses were performed using IBM SPSS Statistics 26.0 software (IBM Corporation, Armonk, NY, USA) with the statistical significance set at *p* ≤ 0.05.

## 3. Results

### 3.1. PVA-BC Composite Morphography

**Figure 3:**
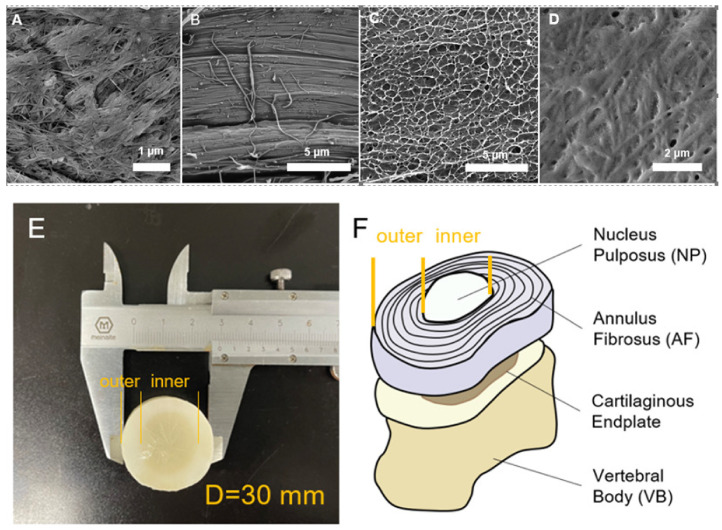
(**A**) SEM image illustrating the nanofibres in a single layer in BC (30.0 kx). (**B**) SEM image of the multiple layers in natural AF (10.0 kx). (**C**) SEM image of the network in crosslinked PVA (20.0 kx). (**D**) SEM image of natural NP (20.0 kx). (**E**) Cross-section of the PVA-BC composite with multiple layers of BC in white, outside, and the inner transparent zone of crosslinked PVA. (**F**) Cross-section of natural IVD.

The nanofibres in BC (Figure 3A) and the concentric layers in natural AF (Figure 3B) are depicted in the scanning electron microscopy (SEM) images. Moreover, the construction of NP (Figure 3D) was mimicked by the crosslinked PVA (Figure 3C). The morphology of a cross section of a PVA-BC specimen fabricated by this method (Figure 3E) closely mimics the natural IVD (Figure 3F). 

### 3.2. Unconfined Compression

**Figure 4:**
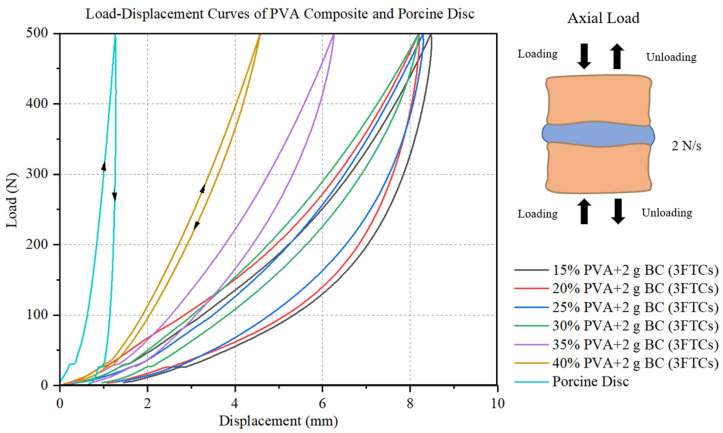
Typical unconfined compression load-displacement hysteresis curves for 15–40% PVA and the porcine disc.

The basic mechanical properties of the PVA-BC composite with respect to the natural IVDs were compared for unconfined compression. Figure 4 illustrates a typical set of stress–strain curves for porcine discs and 15–40% PVA-BC composite containing 2 g BC after 3FTCs. The nonlinear elasticities of the PVA-BC composite and the natural discs are revealed. The curves also feature the different paths of the initial phase of unloading and loading (hysteresis), and the convergence to the starting point, indicating no permanent deformation. The hysteresis can be attributed to the lag in entropic elastic recovery [55,56] and the fluid diffusion into the hydrogel. Another characteristic is the tendency that, at higher concentrations of PVA, the material [57] is harder and less hysteresis lag occurs, featuring left-shifting and closing curves.

### 3.3. Compressive Creep

The compressive stiffness, step displacement, and creep displacement of our PVA-BC composite were calculated (Table 1). Due to the difference between dimensions in our tests and Jesse et al.’s study [54], the corresponding normalised values in Table 2 were used for comparison.

**Figure 5:**
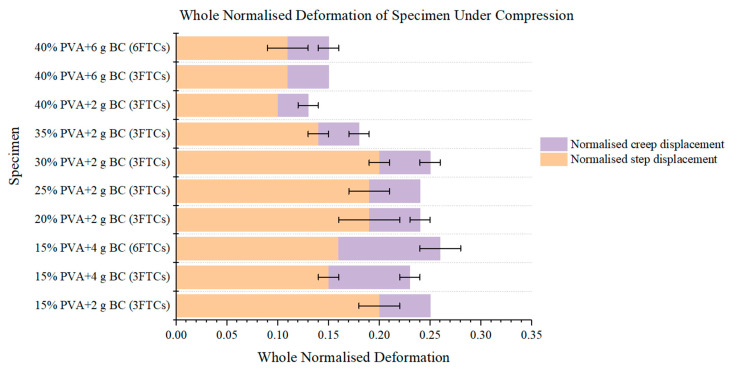
Whole normalised deformation of each PVA-BC composite under compression.

The whole normalised deformation of specimens after preload is depicted in Figure 5. The values of the whole normalised deformation of our PVA-BC composite are close to the values of natural IVDs (0.13), which were 0.08 (±0.00) for normalised step displacement and 0.05 (±0.00) for normalised creep displacement of human L4-L5 according to Jesse et al. [54], indicating the appropriate selection of PVA concentration and fabrication. At higher PVA concentrations, less deformation occurs, which indicates the materials are “harder”. Furthermore, the candidates for novel artificial IVD material are the three for-mulations at 40% PVA (*p* > 0.05), which closely resemble the human L4-L5 (*p* > 0.05).

**Figure 6:**
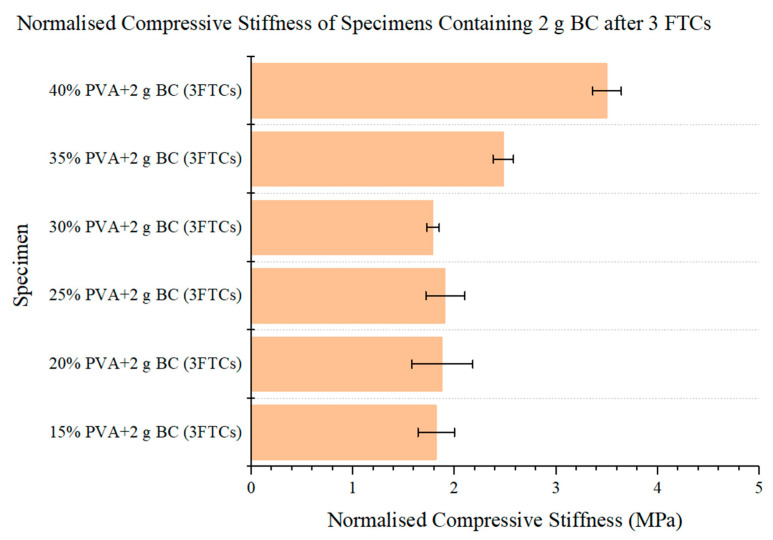
Normalised compressive stiffness of the specimens containing 2 g BC after 3FTCs under compression.

The normalised compressive stiffness of our formulations of PVA-BC composite can be seen from Figure 6, and it differs from natural IVDs. The values of natural IVDs was 9.95 (±3.24) MPa according to Jesse et al. [54], while those of the materials are only between 1.79 and 3.50 MPa.

**Figure 7:**
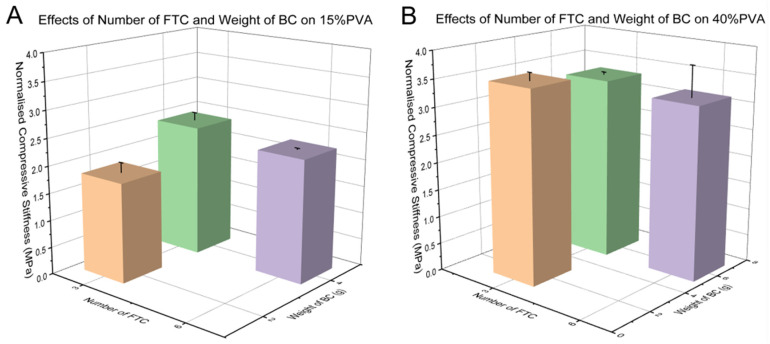
Effects of the number of FTCs and the BC weight on the normalised compressive stiffness of (**A**) 15% and (**B**) 40% PVA.

There was no statistical difference between the specimens containing 2 g BC and 4 g BC in terms of 15% PVA (*p* > 0.05), or between the specimens containing 2 g BC and 6 g BC in terms of 40% PVA (*p* > 0.05). Furthermore, there was no statistical difference between the specimens which underwent 3 FTCs and 6 FTCs containing 15% PVA (*p* > 0.05), or between the specimens which underwent 3 FTCs and 6 FTCs containing 40% PVA (*p* > 0.05). Results can be seen from Figure 7.

Different formulations of the PVA-BC composite exhibited a wide range of normalised compressive stiffness and whole normalised deformation values. Increasing the concentration of the PVA contributes to a higher degree of crystallinity [58,59], and increasing the number of FTCs is associated with the coarsening of the regions of heterogeneity and the increased consolidation of polymer chain entanglement [60]. The BC functions as AF to provide tensile strength. 

### 3.4. Cytotoxicity

The material containing 40% PVA combined with 6 g BC (3FTCs) was used for the cytotoxic analysis as the representative. The absorbance values of every well are presented in Table 3. The cell viabilities of the trial group and the positive control group were calculated with Equation (4), where *A* is the absorbance.

(4) $$Cell\;viability\;\left(\%\right)=\frac{A\;\left(Trial\;group\right)\;or\;A\;\left(Positive\;control\;group\right)}{A\;\left(Blank\;control\;group\right)}\times 100\%$$

According to the standard of ISO 10993-5:2009, a material can be considered to be biocompatible if the value of the cell viability is higher than 70%. The cell viability of the PVA-BC composite is 102.604 (14.136), which is confirmed to have biocompatibility.

## 4. Discussion

With the higher incidence of disc herniation and the increasing complexities of the patients’ needs, traditional fusion and TDR could not provide complete satisfactions for the patients and doctors. An alternative prothesis based on proper selections of structures and biomaterials could help with the reconstruction of spinal biomechanics, and it is also the goal of this study to propose a novel PVA-BC “cushion” that mimics natural IVDs anatomically and functionally.

The complicated multi-chip structures of the existing artificial IVDs (CHARITÉ, Prodisc, and Activ) are problematic, requiring additional assembly by surgeons compared with the integrated cage in fusion operations. The novel, integrated PVA-BC composite mimics the natural structure and mechanics of the IVDs, and makes progress towards the third generation of integrated bionic artificial IVDs.

**Figure 8:**
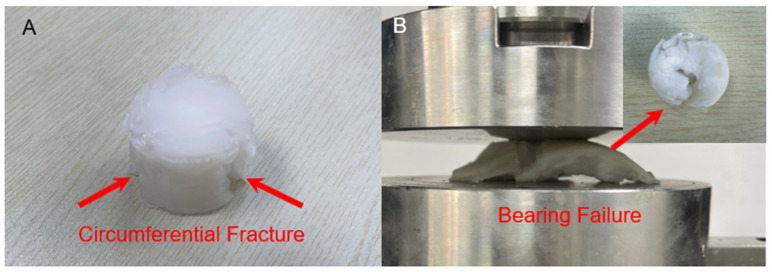
(**A**) 5% PVA + 2 g BC (3FTCs) without compression after manufacturing. (**B**) 10% PVA + 2 g BC (3FTCs) after unconfined compression (a maximum compression of 500 N).

According to our knowledge, the PVA formulations reported in studies range from 7 to 40 wt.% [61,62,63,64,65], and the 5% and 10% PVA were also fabricated in our study in order to characterise the mechanical behaviours. However, after the immersion before the tests, the 5% PVA + 2 g BC (3FTCs) materials fractured circumferentially (Figure 8A) due to their super water absorption and the retention of the low PVA gel concentration, bursting the BC jacket. Similarly, the 10% PVA + 2 g BC (3FTCs) materials incurred bearing failure after compression of 500 N (Figure 8B) because the BC jacket outside cannot bind the inner PVA gel, and thus experiences a large deformation.

Debate continues regarding the duration and load of the in vitro creep tests simulating the physiological conditions. The mechanical parameters of the PVA-BC composite were compared primarily with the natural IVDs from Jesse et al.’s study [54]. They conducted creep tests on animal discs within 1 h, which were 4 h in our study in order to create a strict condition. The magnitudes of the loads are equal, both at 0.48 MPa, corresponding to 750 N human bodyweight divided by a 1560 mm^2^ disc area [66], and the applied load was approximately scaled for the material area as 0.48 × *A*_(*material*)_ (≈300 N). 

We hypothesise that the whole normalised deformation of superior formulations should be equal to Jesse et al.’s results of 1 h, which is stricter while maintaining the same dimensional stability after prolonged compression. The whole normalised deformation results reveal that the three 40% PVA formulations are equal to or slightly larger than the natural IVDs (Figure 5) (*p* > 0.05), illustrating their potentials as candidates for the IVD replacement. Another parameter is the normalised compressive stiffness, while the value varies from the 40% PVA-BC composite to natural IVDs (Figure 6) (*p* < 0.05). Reasons can be attributed to the different structures of natural IVDs and the PVA-BC composite: the former consisted of upper and lower VBs, and the sandwiched IVD (upper right in Figure 4), while the latter only contained the PVA-BC composite, which functioned merely as the IVD. The compressive stiffness calculated based on the sandwich structures of natural IVDs took the stiffness of VBs into account, resulting in a higher value compared with the pure PVA-BC composite [67,68], and was not used as the decisive parameter for the optimal formulation.

Honestly speaking, there is still much room for improvement in terms of the compressive stiffness of our PVA-BC materials toward the natural IVDs. Strengthening strategies include the addition of biopolymers (e.g., polysaccharides and proteins), polymers (e.g., Poly Acrylic Acid (PAA), Polyethylene Glycol (PEG), Polyethylene Pyrrolidone (PVP), Polycaprolactone (PCL) and Polyacrylamide (PAAm)), nanocomposites (e.g., silicon-based, carbon-based and metal oxide-based inorganic materials) and ions (e.g., zwitterionic polymers, common ionic compounds that contain aluminum ions or iron ions), and postprocessing the PVA with curing or annealing [22]. Special attention should be paid in terms of the in situ degradability and dose-dependent cytotoxicity that the additions may bring.

The formulation containing only 40% PVA was not considered by this study, as the addition of BC was necessary for the mimicking of the natural structure of human IVDs, and possessed the advantage of non-degradability by human enzymes [69], which was essential for artificial spinal prostheses in order to ensure the loading durability. Meanwhile, it should be admitted that supplementary studies of only 40% PVA may provide evidence for the assessment of the addition of BC for the achievement of biomechanical mimicry.

Studies on the mechanics of PVA suggest that an increased number of FTCs tends to decrease the amount of swelling of the materials [70,71], and adding BC helps with the tensile strengthening by sharing the load in the composite framework [50,51]. Meanwhile, according to our observation (Figure 7), neither the higher number of FTCs nor more weight of BC seems to have an influence on the normalised compressive stiffness. Further works are still needed to explore the mechanisms of adding BC or increasing the number of FTCs in the improvement of the biomechanical properties.

As for the biocompatibility results, only the material containing 40% PVA combined with 6 g BC after 3FTCs was used for the cytotoxic analysis. Further assessment should be conducted to explore the effects of different concentrations of PVA and BC on cell viability and cell adhesion, and whether the developed PVA-BC composite allows the differentiation of cells toward the neural lineage.

This study was only compared with one prior study, conducted by Jesse et al. [54]. At present, there is a lack of standardised loading protocol in terms of mechanical evaluations of surgical prostheses that are followed by the whole community of biomechanics [72]. To make the results of our study more comparable, a similar testing environment and loading magnitude were replicated merely according to Jesse’s study in order to support a proper comparison. Further studies should be conducted with the aim of a direct comparison between artificial implants and natural discs using the same biomechanical tests.

In order to address the problem of fixation, the protein-based biological adhesives—containing catechol amino acid (DOPA), phosphonate, and divalent metal ions that contribute to strong adhesion on wet surfaces [73,74,75]—have been considered as bonded materials at the interfaces. The advantage of not using metal endplates is that they do not create imaging artefacts, making them more compatible with follow-up imaging such as MRI [15]. An additional advantage of the integrated design is that traditional endplates could limit the ROM by impinging at a certain angle during flexion/extension or lateral bending. Without endplates, the ROM is only limited by the viscoelasticity of the material and the surrounding ligaments, muscles, and soft tissue, which mimics the physiological condition of a natural IVD. This leads to a shrinkage in the structure, which might create an instability, and there might be a risk of damaging the soft material during the operation process. Moreover, the implanted material may suffer from fretting wear at the bone surfaces after implantation. The risk of reduced osseous integration between the vertebrae and the implant is another disadvantage, while some surface treatments, such as plasma etching or hydroxyapatite coating, can be added to promote bone ingrowth [15].

Further fatigue tests are also required because the low normalised compressive stiffness and large step displacement of the PVA-BC composite imply that it is softer than the natural IVDs with comparable whole normalised deformation. Multiple reciprocating loading could facilitate an assessment of the load failure and failure form during the fatigue tests.

## 5. Conclusions

The 40% PVA combined with 2 g BC (3FTCs), 6 g BC (3FTCs), or 6 g BC (6FTCs) formulations with the bionic structural design may have potential in the application of TDR. The unconfined compression confirmed that the PVA-BC composite has similar nonlinear elasticity and hysteresis to natural IVDs. The normalised compressive stiffness and whole normalised deformation were compared with natural IVDs via compressive creep, showing acceptable mimicry and ensuring dimensional stability. Combined with the cell viability results, this novel bionic structure with its formulation of the PVA-BC composite has excellent biocompatibility, making it a suitable choice as a material in third-generation artificial IVDs that mimic the natural IVDs anatomically and mechanically.

The results of this investigation address the research gaps regarding the mechanical properties of bionic PVA-BC composites, especially concerning human IVDs. The results provide a solid foundation for future works in biomimetic material design and fabrication, advancing superior applications of PVA-BC composite towards implantable spinal prostheses which are intended to restore IVD biomechanics. The novel integrated IVD design has potential for animal trials and ultimate clinical use.

## Figures and Tables

**Table 1:** Mean (standard deviation) for measured mechanical parameter values for each PVA-BC composite.

Specimen	Compressive Stiffness (N/mm)	Step Displacement (mm)	Creep Displacement (mm)
15% PVA+2 g BC (3FTCs)	64.23 (6.39)	3.925 (0.36)	1.035 (0.05)
15% PVA+4 g BC (3FTCs)	85.66 (4.96)	2.94 (0.16)	1.57 (0.25)
15% PVA+4 g BC (6FTCs)	79.90 (0.61)	3.12 (0.03)	1.96 (0.37)
20% PVA+2 g BC (3FTCs)	66.46 (10.46)	3.83 (0.64)	1.07 (0.25)
25% PVA+2 g BC (3FTCs)	67.62 (6.77)	3.76 (0.36)	0.97 (0.02)
30% PVA+2 g BC (3FTCs)	63.06 (2.01)	3.99 (0.14)	0.99 (0.12)
35% PVA+2 g BC (3FTCs)	87.52 (3.59)	2.85 (0.13)	0.82 (0.18)
40% PVA+2 g BC (3FTCs)	123.48 (4.79)	2.04 (0.08)	0.53 (0.18)
40% PVA+6 g BC (3FTCs)	117.25 (1.32)	2.15 (0.02)	0.70 (0.01)
40% PVA+6 g BC (6FTCs)	111.77 (20.15)	2.28 (0.42)	0.81 (0.16)

Values are presented as mean over (standard deviation).

**Table 2:** Mean (standard deviation) for normalised mechanical parameter values for each PVA-BC composite.

Specimen	Normalised Compressive Stiffness (MPa)	Normalised Step Displacement	Normalised Creep Displacement
15% PVA+2 g BC (3FTCs)	1.82 (0.18)	0.20 (0.02)	0.05 (0.00)
15% PVA+4 g BC (3FTCs)	2.42 (0.14)	0.15 (0.01)	0.08 (0.01)
15% PVA+4 g BC (6FTCs)	2.26 (0.02)	0.16 (0.00)	0.10 (0.02)
20% PVA+2 g BC (3FTCs)	1.88 (0.30)	0.19 (0.03)	0.05 (0.01)
25% PVA+2 g BC (3FTCs)	1.91 (0.19)	0.19 (0.02)	0.05 (0.00)
30% PVA+2 g BC (3FTCs)	1.79 (0.06)	0.20 (0.01)	0.05 (0.01)
35% PVA+2 g BC (3FTCs)	2.48 (0.10)	0.14 (0.01)	0.04 (0.01)
40% PVA+2 g BC (3FTCs)	3.50 (0.14)	0.10 (0.00)	0.03 (0.01)
40% PVA+6 g BC (3FTCs)	3.32 (0.04)	0.11 (0.00)	0.04 (0.00)
40% PVA+6 g BC (6FTCs)	3.16 (0.57)	0.11 (0.02)	0.04 (0.01)

Values are presented as mean over (standard deviation).

**Table 3:** Absorbance and cell viability in the cytotoxicity tests.

Group	Trial group(MEM + PVA-BC)	Blank control group(MEM)	Positive control group (MEM+DMSO)
**Absorbance**	0.847 (0.117)	0.826 (0.070)	0.304 (0.030)
**Cell viability (%)**	102.604 (14.136)	-	36.839 (3.639)

Values are presented as mean over (standard deviation).
